# Artificial visual perception neural system using a solution-processable MoS_2_-based in-memory light sensor

**DOI:** 10.1038/s41377-023-01166-7

**Published:** 2023-05-05

**Authors:** Dayanand Kumar, Lana Joharji, Hanrui Li, Ayman Rezk, Ammar Nayfeh, Nazek El-Atab

**Affiliations:** 1 Smart, Advanced Memory Devices and Applications (SAMA) Laboratory, Electrical and Computer Engineering Program, Computer Electrical Mathematical Science and Engineering Division, King Abdullah University of Science and Technology (KAUST), Thuwal, 23955 Kingdom of Saudi Arabia; 2 Department of Electrical Engineering and Computer Science, Khalifa University, Abu Dhabi, 127788 United Arab Emirates

**Keywords:** Optical data storage, Optical sensors

## Abstract

Optoelectronic devices are advantageous in in-memory light sensing for visual information processing, recognition, and storage in an energy-efficient manner. Recently, in-memory light sensors have been proposed to improve the energy, area, and time efficiencies of neuromorphic computing systems. This study is primarily focused on the development of a single sensing-storage-processing node based on a two-terminal solution-processable MoS_2_ metal–oxide–semiconductor (MOS) charge-trapping memory structure—the basic structure for charge-coupled devices (CCD)—and showing its suitability for in-memory light sensing and artificial visual perception. The memory window of the device increased from 2.8 V to more than 6 V when the device was irradiated with optical lights of different wavelengths during the program operation. Furthermore, the charge retention capability of the device at a high temperature (100 °C) was enhanced from 36 to 64% when exposed to a light wavelength of 400 nm. The larger shift in the threshold voltage with an increasing operating voltage confirmed that more charges were trapped at the Al_2_O_3_/MoS_2_ interface and in the MoS_2_ layer. A small convolutional neural network was proposed to measure the optical sensing and electrical programming abilities of the device. The array simulation received optical images transmitted using a blue light wavelength and performed inference computation to process and recognize the images with 91% accuracy. This study is a significant step toward the development of optoelectronic MOS memory devices for neuromorphic visual perception, adaptive parallel processing networks for in-memory light sensing, and smart CCD cameras with artificial visual perception capabilities.

## Abstract (web summary)

A smart memory device that can sense and compute using an MoS_2_ charge-trapping layer is demonstrated for application in neuromorphic vision.

## Graphical abstract

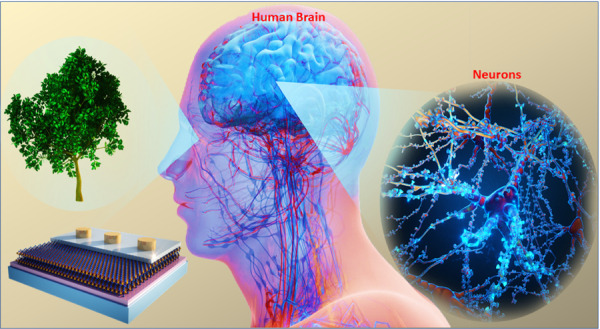

## Introduction

In this modern era, artificial intelligence and the Internet of Things have led to the rapid expansion of sensory nodes, which produce an increasing volume of raw data^1–5^. In general systems, analog sensory data are transformed into digital data and further transferred to other units to perform computational tasks^6–12^. Nevertheless, in the conventional von Neumann architecture, the separation of sensory terminals and computing units consumes high power while delaying data access with hardware redundancy^9,13^. Alternatively, advanced technology-based in-memory sensing and computing are anticipated to improve the response time as well as the area and energy efficiency. These implementations are advantageous in minimizing delays owing to data movement between different units, especially in sensor-rich platforms and applications, such as autonomous driving, self-driving microrobots, and real-time video analysis^14,15^.

**Fig. 1:**
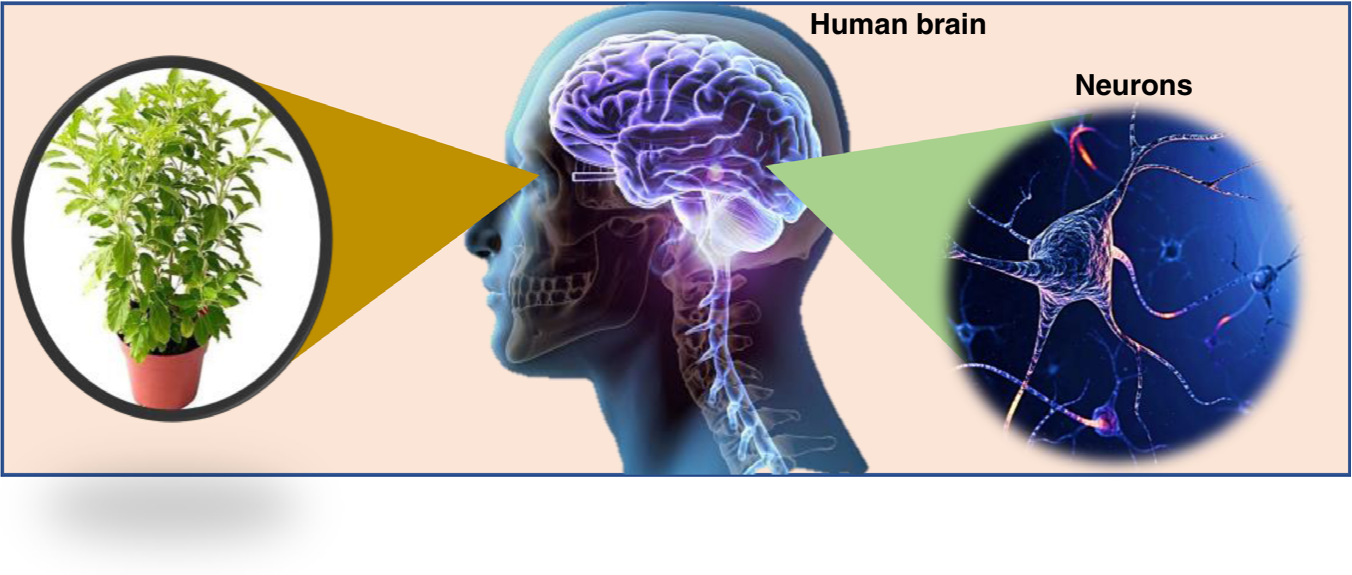
Schematic of the human visual-perception process

Recently, two-dimensional (2D) transition-metal dichalcogenide (TMD)-based optoelectronic devices have been broadly considered because of their attractive optical and electrical features^16^. Owing to their fascinating features, these devices have shown promising applications in photovoltaics, artificial visual perception, photosensors^17,18^, and neuromorphic synaptic devices^19^. Furthermore, advanced designs in logic-in-sensor, in-memory computing, and in-memory light sensing have been proposed for novel 2D materials^20^. Visual perception is one of the vital human senses where the brain decodes what the eyes see. The human eye receives more than 80% of information through sight. The visual-perception process in the brain is illustrated in Fig. 1, wherein the human eye receives light from an external source. This light is focused on the retina of the eye, which captures an image of the visual stimuli. Nerve cells present in the retina function as photoreceptors that convert light into electrical impulses. These impulses move from the optic nerve to the visual cortex at the back of the human brain. Therefore, novel material-based devices for optical sensing and online data processing are of great significance in artificial intelligence applications and in-memory light sensing.

2D TMD materials can be grown via chemical vapor deposition (CVD) or transferred using physical and chemical exfoliation methods. Particularly, the mechanical exfoliation method has enabled the fabrication of high-quality TMDs, such as MoS_2_, WS_2_, ZrS_2_, MoSe_2_, NbSe_2_, TiS_2_, TaS_2_, and WSe_2,_ for several applications^21^. Lee et al. reported the development of single- and multilayer MoS_2_ and WSe_2_ based on mechanical exfoliation^21–23^. An et al. reported that TMD-based nanosheets, such as MoSe_2_, MoS_2_, WS_2_, and WSe_2_, could be obtained by the exfoliation method using a high-power laser^24^. The main disadvantage of this approach is that a large amount of material is needed to obtain the desired nanosheets and large-sized monolayers. On the one hand, although CVD provides more extensive coverage of the 2D material, its main disadvantage is the high thermal budget required, thereby making it incompatible with back-end-of-line processing or wearable and flexible electronics. On the other hand, the spin coating of 2D materials makes it possible to obtain a large density of the material at low temperatures, with the main disadvantage being a lack of control and uniformity. Nevertheless, Matsuba et al. have shown that it is possible to achieve full monolayer coverage using the spin-coating technique, which makes solution processing very promising^25^.

In this study, MOS memory architecture is examined using a light-sensitive 2D material-based charge-trapping layer. This makes the solution-processable technology promising as a low-cost and low-thermal-budget coating technique. This is necessary when dealing with highly sensitive optical components on a chip and is critical for allowing back-end-of-line compatibility. Alongside other 2D materials, MoS_2_ is very attractive owing to its transition from an indirect bandgap of 1.2 V to a direct bandgap of 1.8 eV. This is sufficient to balance the limitations of zero-bandgap graphene for use in electronic devices^25^. To boost the programming and erase currents on/off ratio, the graphene layer was replaced with MoS_2_ as a charge-storage layer in a flash memory device^26^. A report showed that MoS_2_ (especially a monolayer) was highly sensitive to charge presence^27^. Thus far, few studies have reported on MOS_2_-based MOS memory devices. However, their poor performance in terms of narrow memory windows at low operating voltages, poor endurance, and insufficient charge storage capability have restricted their use in artificial intelligence^28–33^. Hence, further improvements are required in MoS_2_-based MOS memory for artificial intelligence systems.

Recently, researchers have reported that the threshold voltage of a transistor or memory can be shifted when the optical light is illuminated. The memory window of a transistor was modulated using different light wavelengths^34–36^. This phenomenon is primarily due to the application of photoinduced charge carriers. The threshold voltage shifting due to incident light with bias voltage confirms the possibility of using photogenerated carriers in in-memory light sensors^37–39^. In fact, the change in the threshold voltage under light illumination widens the application spectrum from electrical to optical systems and light-sensing devices. To the best of our knowledge, no prior work on light-sensitive MOS memory devices has been reported to be useful for application in in-memory light sensing and artificial intelligence systems. In this study, we present a MoS_2_-based MOS capacitor that is highly suitable for in-memory light sensors and artificial visual perception. It is noteworthy that the demonstrated MOS memory devices use a similar structure as the Nobel Prize-winning charge-coupled devices (CCD) in CCD cameras, which makes this study a significant step toward the development of smart CCD cameras with artificial visual perception capabilities. The memory window of the MOS memory increased from 2.8 V to more than 6 V when the optical light of 400 nm wavelength was incident on the device during the programming voltage of +6/−6 V. The larger shift in the threshold voltage with a decreasing light wavelength confirmed that charges were trapped at the Al_2_O_3_/MoS_2_ interface and in the MoS_2_ layer. The convolutional neural network was designed to demonstrate the optical sensing and electrical programming capabilities of the device. This study represents a significant step toward the development of an optical MOS memory for neuromorphic visual perception and in-memory light sensing. Table 1 shows a comparison between previously reported MoS_2_-based memory devices and those used in this study^29,34,40–46^.

**Table 1:** Comparison between previously reported MoS_2_-based charge trapping memory (CTM) and our study

MoS_2_ based CTM	Operating condition	Endurance	Retention	Ref.
MoS_2_/high-k	+26 V/−26 V/200 ms	120 cycles	2000 s at RT	^29^
MoS_2_/high-k	+12 V/100 μs, −10 V/10 μs	8000 cycles	10000 s at RT	^71^
Al_2_O_3_/MoS_2_/Al_2_O_3_	+60 V/−60 V	1000 cycles	10 days at RT	^72^
MoS_2_/high k (TTO)	+4 V/−4, 1 ms	1000 cycles	1000 s at RT	^34^
MoS_2_/CrPS_4_	+30 V/−30 V	625 cycles	-------	^73^
HfO_2_/MoS_2_/SiO_2_	+16 V/−16, 1 s	550 cycles	10000 s at RT	^74^
GDY/MoS_2_	−80 V/+80 V	3000 cycles	10000 s at RT	^75^
GBM3	+16 V/−16 V, 100 μs	-----------	1200 s at RT	^70^
MoS_2_/h-BN	+5 V/−5 V, 100 ms	10^5^ cycles	10^8^ s at RT	^76^
**Al**_**2**_**O**_**3**_**/MoS**_**2**_**/Al**_**2**_**O**_**3**_	**+6** **V/**−**6** **V, 1** **μs**	**Electrically programmed/erased: 10**^**6**^ **cycles** **Optically programmed/electrically erased: 1000 cycles** **Wavelength: 600** **nm, 550** **nm, 500** **nm, 450** **nm, 400** **nm**	**10 years at 100°C**	**This work**

## Results

**Fig. 2:**
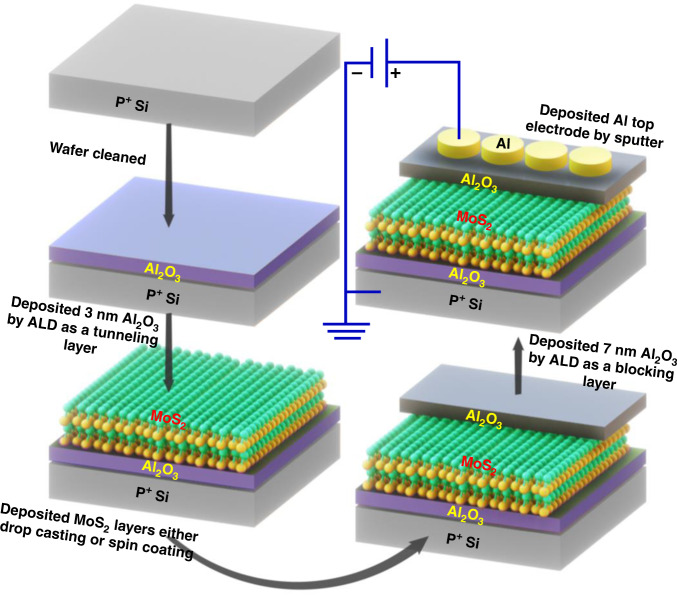
Schematic of the fabrication process of the light-sensitive MOS memory devices

**Fig. 3:**
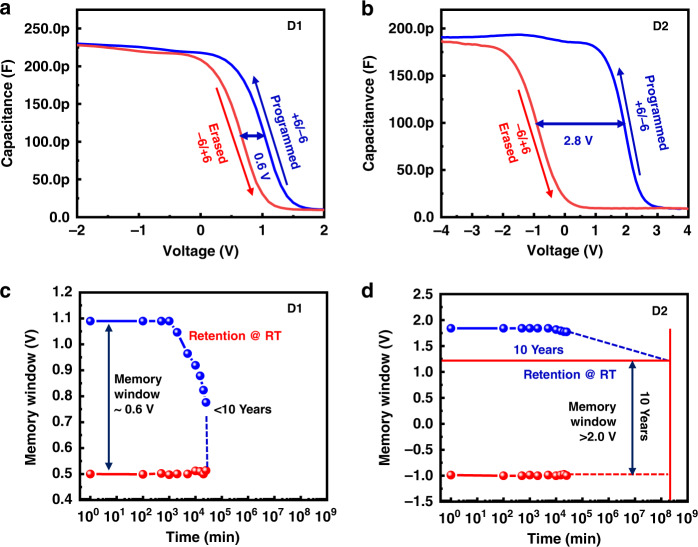
Effect of spin coating *vs*. drop casting of MoS_2_ flakes on the memory electrical performance. **a**, **b** High frequency (80 kHz) C–V curves of the D1 and D2 devices with a memory window of 0.6 V and 2.8 V, respectively. **c**, **d** Retention tests of devices D1 and D2

The complete fabrication process of the light-sensitive MOS devices is shown in Fig. 2. It includes solution-processed MoS_2_ sandwiched between atomic-layer-deposited Al_2_O_3_ layers in a charge-trapping MOS memory architecture. The charging properties and retention tests of the coated MoS_2_-based Al/Al_2_O_3_/MoS_2_ (spin-coated)/Al_2_O_3_/P^+^-Si (D1) and drop-cast MoS_2_-based Al/Al_2_O_3_/MoS_2_ (drop-cast)/Al_2_O_3_/P^+^-Si (D2) devices were measured, as shown in Fig. 3. The C–V curves of devices D1 and D2 were analyzed, as shown in Fig. 3a, b, respectively. Both devices were operated with a sweep voltage of +6/−6 V (sweep rate 0.01 mV/s) in the forward and reverse conditions at an 80 kHz frequency. The obtained memory window for both devices was approximately 0.6 and 2.8 V, respectively. The retention tests were conducted at room temperature, as shown in Fig. 3c, d. The D1 device showed retention degradation after 10^3^ min, while device D2 displayed highly stable retention of at least 10^4^ min. It was predicted to have a 10-year lifetime while maintaining a memory window of more than 2.8 V. The large memory window (>2 V) and excellent predicted retention (>10 years) in the D2 device were due to the trapping of a significant number of electrons in the Al_2_O_3_/MoS_2_ interface and MoS_2_ layer compared to the D1 device. This significant improvement was also a result of the high density of the MoS_2_ layers in the D2 device. This is explained with the help of scanning electron microscopy (SEM) analysis in the sections below.

**Fig. 4:**
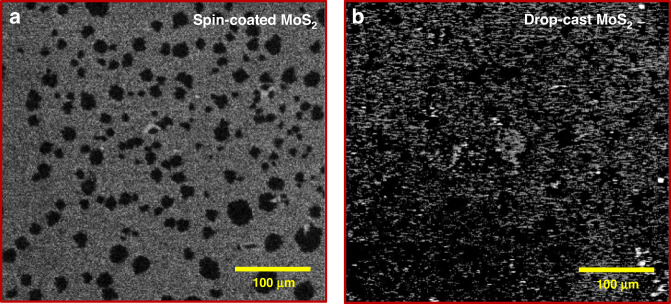
Effect of spin coating *vs*. drop casting techniques on MoS_2_ flakes surface coverage. **a** SEM image of MoS_2_ flakes using the spin-coating technique showing a lower density of flakes. **b** SEM image of MoS_2_ flakes using the drop-casting technique showing the large density of flakes

To check the difference between the spin-coated and drop-cast MoS_2_ flakes of the D1 and D2 samples, the surface morphologies of both samples were analyzed, as shown in Fig. 4a, b, respectively. The SEM image shows that the spin-coated MoS_2_ sample includes a smaller number of flakes with a poor density (Fig. 4a). Owing to the smaller number of flakes, the MoS_2_ layer trapped few charges in the D1 device. Hence, the D1 device illustrated a small memory window and poor retention at room temperature, as shown in Fig. 3a, c. We believe that the large number of flakes and high density of MoS_2_ contributed to the trapping of more charges at the Al_2_O_3_/MoS_2_ interface and MoS_2_ layer in the D2 device. Consequently, the D2 device showed a wider memory window (>2.8 V) and high retention of at least 10 years without any degradation, as portrayed in Fig. 3b, d, respectively. Because the D2 device showed improved performance compared to the D1 device, further experiments were conducted on the D2 device only.

**Fig. 5:**
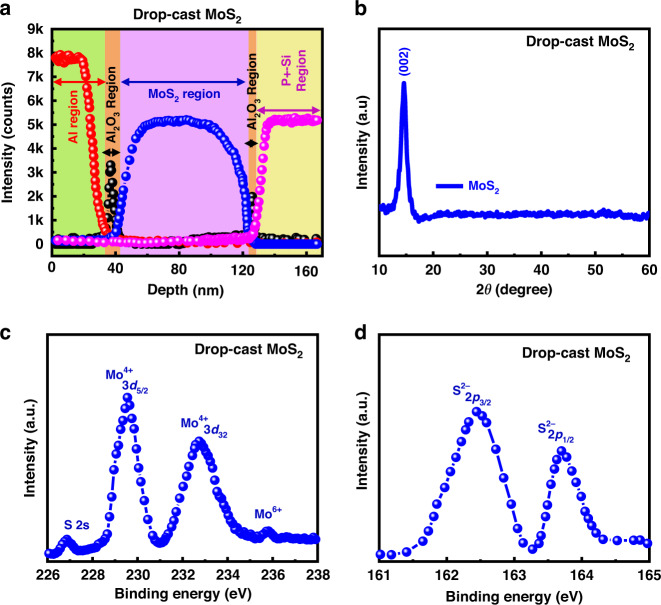
Drop casted MoS_2_ material characterization. **a** The SIMS depth profile of the D2 device, **b** XRD spectra of the drop-cast MoS_2_ film, **c** Mo 3d and S 2s, and **d** S 2p XPS peaks of the drop-cast MoS_2_ film

To validate each layer in the D2 device, secondary ion mass spectrometry (SIMS) analysis was performed, as shown in Fig. 5a. The device was etched from the Al top electrode to the bottom P^+^-Si, which enabled the measurement of the depth profile of the device. Each layer is clearly shown, confirming the thicknesses of the blocking oxide Al_2_O_3_ layer (~7 nm), charge trapping MoS_2_ layer (~70 nm), and tunnel oxide Al_2_O_3_ layer (~3 nm).

The crystal structure of drop-cast MoS_2_ was measured using X-ray diffraction (XRD), as shown in Fig. 5b. XRD diffraction confirmed a sharp and narrow peak at approximately 14.5°, which was recognized as the (002) plane of MoS_2_^47–49^. The XRD spectra of the sample were matched with previously reported data for a hexagonal MoS_2_ thin film (JCPDS:37-1492). After matching the XRD spectrum of the sample with that of JCPDS, it was confirmed that there was no shift in the 2θ value for the MoS_2_ thin film. The XRD spectra of the sample also suggested that the (002) plane was highly oriented. To explain the surface chemical states and coordination geometry of the drop-cast MoS_2_ flakes, X-ray photoelectron spectroscopy (XPS) analysis was carried out to determine the binding energies of MoS_2_, as shown in Fig. 5c, d, respectively. In Fig. 5c, Mo 3d shows three binding energy peaks located at approximately 226.8, 229.4, and 232.8 eV. The binding energy peaks at 229.4 and 232.8 eV are due to the doublets of Mo 3d_5/2_ and Mo 3d_3/2_, respectively^50–56^. The S 2 s peak (weak sulfur), located at a binding energy of 226.8 eV (Fig. 5c), and divalent sulfide ion (S^2−^) peaks with binding energies of approximately 162.4 and 163.7 eV were allocated to 2p_1/2_ and 2p_3/2_, respectively, as shown in Fig. 5d. Additionally, from the XPS analysis, we found a small peak located at a higher binding energy of 235.6 eV, which is attributed to Mo^6+^. This shows that the Mo edges in the drop-cast MoS_2_ were oxidized during the deposition of the MoS_2_ film. This suggests that the interfacial oxidation between MoS_2_ and the Al_2_O_3_ layer is due to the deposition of MoS_2_^56–59^. Conclusively, XPS analysis confirmed that the drop-cast MoS_2_ layer had a hexagonal structure^60^.

**Fig. 6:**
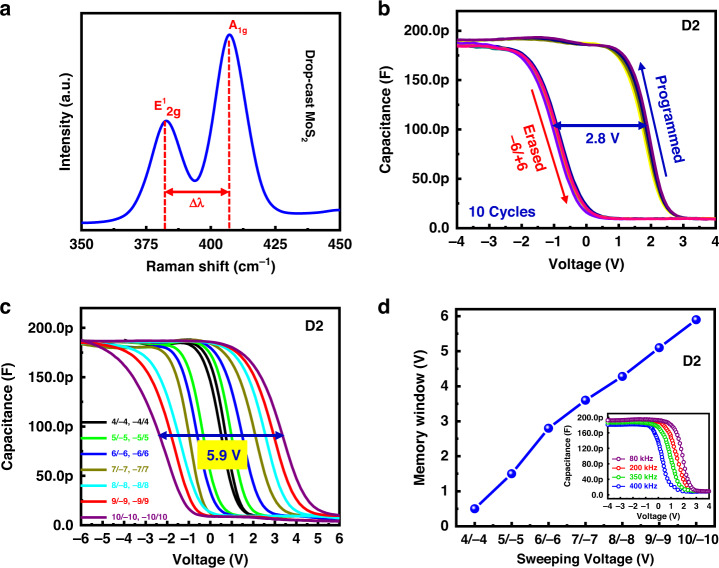
MoS_2_ structural characterization and the resulting charge trapping memory electrical performance. **a** Raman spectrum of the drop-cast MoS_2_ layer, **b** C–V characteristics of the D2 device for 50 repeated programmed and erased cycles with a frequency of 80 kHz, **c** C–V characteristics of the device with sweeping voltages of +4/−4 to +10/−10 in the programmed and erased conditions, and **d** memory window of the device with sweeping voltages (inset shows the frequency-dependent C–V curve of the D2 device)

The Raman spectrum of the drop-cast MoS_2_ film is shown in Fig. 6a. It shows two characteristics of the MoS_2_ Raman peaks. The E^1^_2g_ and A_1g_ modes are located at approximately 380 cm^−^^1^ and 407 cm^−^^1^, respectively. Both Raman peaks (380 cm^−^^1^ and 407 cm^−^^1^) are separated by Δλ ~ 25 cm^−^^1^, which is considerably higher than that of the previously reported monolayer and several MoS_2_ film layers^61–63^. This result suggests that our sample has many layers of MoS_2_, which is consistent with the SIMS analysis of the MoS_2_ film (Fig. 5a)^61,62^. To explain the memory window of the D2 device, the C–V measurements were taken at room temperature with a frequency of 80 kHz, as shown in Fig. 6b. The device was measured with a sweeping voltage of +6/−6 in forward and backward conditions for 50 repetitive programmed and erased cycles. The memory window of the device was measured using the difference in flat-band voltages (V_FB_) between the programmed and erased states. The memory window of the device was observed at approximately 2.8 V when sweeping the voltage at +6/−6 in the forward and backward states. Furthermore, we checked the memory window of the device by increasing the sweeping voltages from +4/−4 to +10/10 in the programmed and erased conditions, as shown in Fig. 6c. The memory window of the device was increased by increasing the sweeping voltages, and the maximum was approximately 5.9 V at +10/−10 V, showing an excellent charge trapping effect (Fig. 6d). The trapped charge density in the D2 device can be calculated using the following equation^64–68^.

1 $${{\rm{N}}}_{{\rm{t}}}={{\rm{C}}}_{{\rm{t}}}\times \varDelta {{\rm{V}}}_{{\rm{t}}}/{\rm{q}}$$

where C_t_ is the capacitance of the device per unit area, ΔV_t_ is the memory window of the device at V_FB_, and q is the elementary charge. Using the above equation, the calculated trapped charge density of the D2 device is approximately 9.2 × 10^13^cm^−^^2^.

It was confirmed that the D2 device showed a considerable improvement in the memory window under low operating voltages. This improvement in the D2 device is due to the large number of electrons trapped at the Al_2_O_3_/MoS_2_ interface and MoS_2_ layer. To confirm the interface trapping in the D2 device, we measured the frequency-dependent C–V characteristics of the device, as shown in the inset of Fig. 6d. The capacitance of the D2 device upon accumulation decreased with increasing frequency. This confirmed the existence of interface states at the MoS_2_/Al_2_O_3_ interface^69^.

**Fig. 7:**
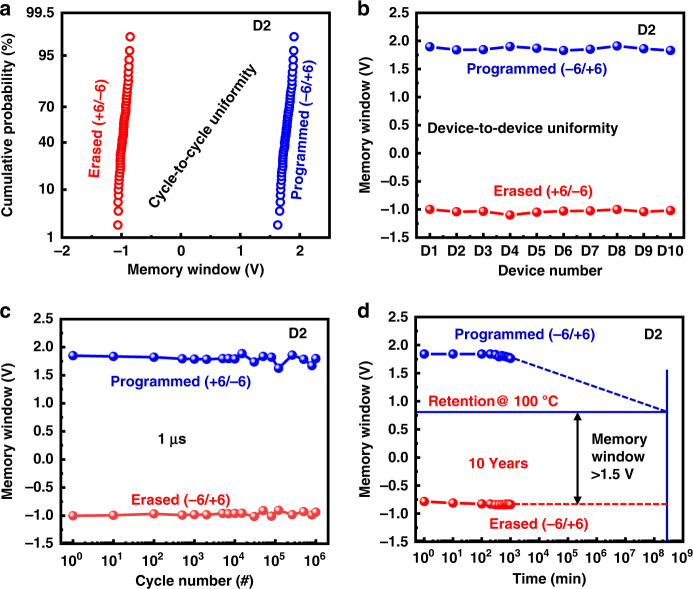
Reliability characteristics of MoS_2_ based memory. **a** Cycle-to-cycle uniformity of the D2 device, **b** device-to-device uniformity of the 10 devices that were chosen randomly, **c** long-term endurance cycles of the device, and **d** high-temperature retention test of the device

The cycle-to-cycle uniformity of the device was calculated with operating voltages of +6/−6 during the forward and backward states, as shown in Fig. 7a. The device shows that the memory window was highly stable under both programming and erasing conditions. The device-to-device stability in both the programmed and erased states of the D2 device was analyzed for ten randomly chosen devices, as portrayed in Fig. 7b. This figure confirms that the D2 device showed excellent stability under both programming and erasing conditions for all 10 devices. To confirm the long-term stability in both the programming and erasing states, the endurance of the device was measured with programming and erasing voltages of +6/−6 and −6/+6, respectively, as shown in Fig. 7c. The device exhibited a highly stable endurance for at least 10^4^ cycles with a memory window of approximately 2.8 V. The high-temperature retention test of the device is shown in Fig. 7d. This device can sustain its programming and erase states for at least 10^3^ minutes and is predicted to have more than 10 years of life with a memory window of more than 1.5 V at a temperature of 100 °C. These excellent features make them promising for in-memory light sensing and artificial intelligence systems.

**Fig. 8:**
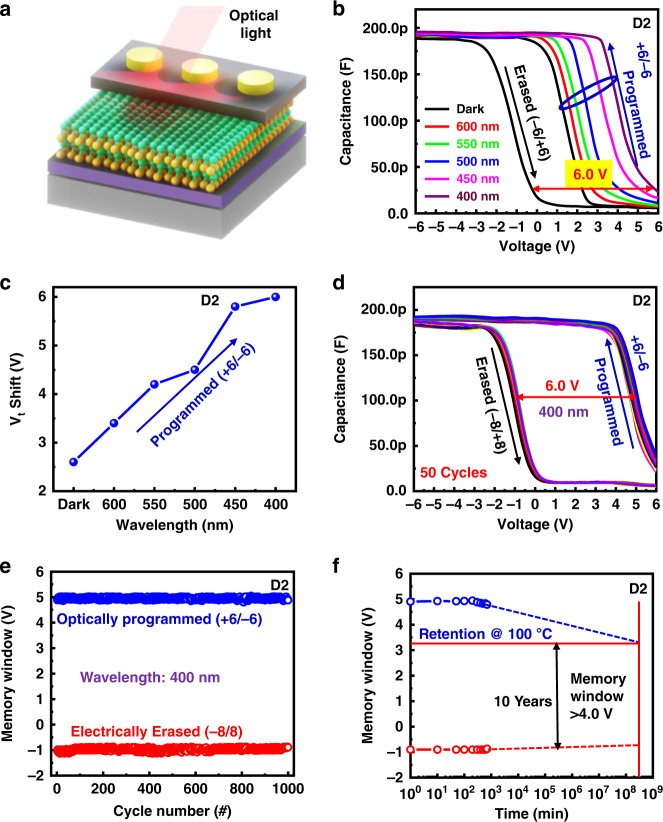
Optical charaterization of the MoS_2_ based in-memory sensor. **a** Schematic of the device with optical light illumination, **b** C–V curves of the device using different optical light wavelengths from 600 to 400 nm with an interval of 50 nm, **c** wavelength-dependent threshold voltage of the device, **d** repeatability of C–V curves for 50 continuous cycles with optical programming (+6/−6) and electrically erasing (−8/+8), **e** optically programmed and electrically erased endurance of the device with illumination at a wavelength of 400 nm, and **f** high-temperature retention stability of the device

In addition to the electrical features, we also measured the light-sensing features of the D2 device using irradiation with different light wavelengths, as portrayed in Fig. 8. A schematic of the device is shown in Fig. 8a. The optical characteristics of the device were studied by illuminating it with light. The C–V characteristics of the device under programming and erasing conditions are shown in Fig. 8b. The optical characteristics of the device were measured using different light wavelengths from 600 to 400 nm with a tunable light source (Newport Corporation). When the device was in dark conditions, the memory window of the device was observed at approximately 2.8 V. When the light was turned on for 2 s on the device with 600 nm wavelength (intensity: 2 mW cm^−^^2^) during the programming condition of +6/−6 V, the threshold voltage increased from 2.5 to 3.3 V.

This means that owing to the illumination of the optical light, the device trapped more electrons compared to the dark condition. Furthermore, we examined the effect of different light wavelengths from 550 to 400 nm with a 50 nm interval using a similar programming voltage, intensity, and illumination time. We recorded a tremendous shift in the threshold voltage from 3.3 V to more than 6 V. The maximum shift in the threshold voltage was observed at a wavelength of 400 nm. Thus, it was confirmed that the MoS_2_ layer trapped more electrons at a wavelength of 400 nm^70–72^. When smaller light wavelengths (from 600 to 400 nm) are irradiated onto the sample, more carriers are generated in the MoS_2_^73,74^, leading to an increased probability of the device’s charge trapping. Therefore, a shift in V_th_ (as shown in Fig. 8c) and a larger memory window are observed when smaller light wavelengths are used. The D2 device shows that the threshold voltage increases with a decrease in the light wavelength. By contrast, the memory window of the device increases with a decrease in the wavelength. The optically programmed (+6/−6) state using a light illumination (400 nm) of 2 s and the electrically erased (+8/−8) state for 50 repeatable programmed and erased cycles are shown in Fig. 8d. The memory window of the device was greater than 6 V. The high memory window of the device is due to the large number of trap states in the MoS_2_ layer and Al_2_O_3_/MoS_2_ interface in the D2 device^75^. This corresponds with Choi et al., who reported that when optical light with a specific wavelength was incident onto the device, the electrons were trapped at the interface of dielectric/MoS_2_ and inside the MoS_2_ layer^76^. The optically programmed and electrically erased endurance of the device is shown in Fig. 8e. The device displayed a high and stable memory window during optical programming and electrical erasing for at least 1,000 cycles without any degradation. A high-temperature retention test of the device is shown in Fig. 8f. This device is able to maintain both states (programmed and erased) for at least 10^3^ min and predict 10 years at 100 °C with a memory window of 4 V. These excellent results confirm that the device is capable of in-memory light sensing.

## Discussion

**Fig. 9:**
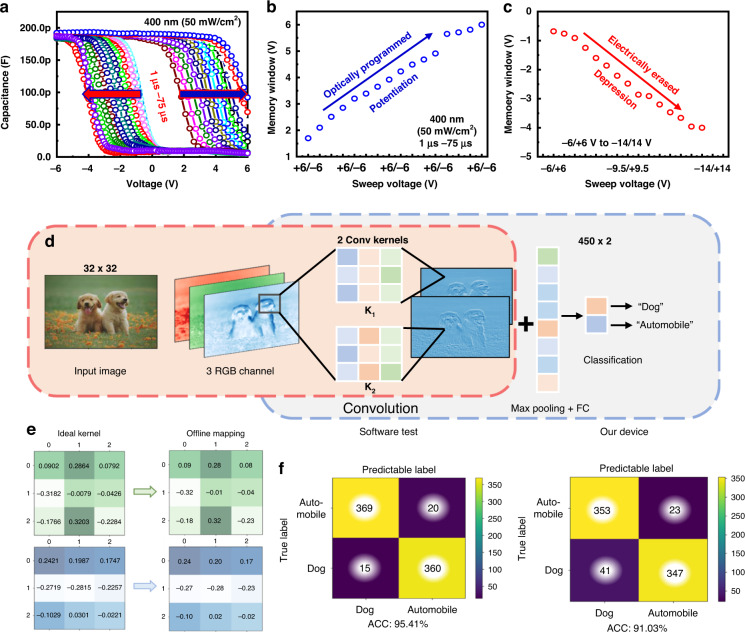
Application of the in-memory optical sensor in artificial visual perception. **a** C–V curve of the D2 device using an optical light of 50 mW cm^−^^2^ (400 nm) from 1 to 75 µs with an interval of 5 µs; **b** Memory window of the device, which is optically programmed; **c** Memory window of the device that is electrically erased; **d** A small CNN model is used to make a binary classification over the CIFAR-10 dataset; **e** The kernels (left) are obtained from the ideal software test. The offline mapping kernels (right) are transferred from the corresponding ideal kernel values by illuminating and programming the device. **f** The confusion matrix of the test results for 764 images in the CIFAR-10 dataset. The yellow-colored diagonal elements in the matrix represent the correctly identified cases

To validate the potential of the MoS_2_-based in-memory light sensor for artificial visual perception, we analyzed the synaptic features of the optical D2 device. Optical light (400 nm) with an intensity of 50 mW cm^−^^2^ was used for programming, while the device was erased electrically. The C–V curves of the device, when optically programmed and electrically erased, are shown in Fig. 9a. For the programming condition, the optical light was irradiated from 1 to 75 µs with an interval of 5 µs, and the device showed a threshold voltage shift toward the positive side with time. It is noteworthy that the C–V curve was measured immediately after irradiation. For the erasing condition, sweeping voltages from −6/+6 V to −14/+14 V with an interval of 0.5 V were used, and the threshold voltage was shifted to the negative side during erasing. In fact, the longer the pulse width is, the greater the probability of the generated carriers being trapped within the trapping sites of MoS_2_ and/or at the interface with Al_2_O_3_^70^. Owing to the larger charge trapping, a threshold voltage shift was observed in the MOSCAP-based memory. The memory windows of the D2 device during optical programming (potentiation) and electrical erasing (depression) are shown in Fig. 9b, c, respectively. We observed potentiation and depression with optical programming and electrical erasing, respectively, as shown in Fig. 9b, c, respectively. A small convolutional neural network (CNN) was designed to demonstrate the optical sensing and electrical programming abilities of the D2 device. We extracted images from the Canadian Institute For Advanced Research (CIFAR)-10 dataset to make a simple binary image recognition^77^, wherein the objects “dog” and “automobile” were chosen as the classification tasks. As shown in Fig. 9d, the CNN structure comprises a convolutional layer with two 3×3 kernels, a max pooling layer for feature extraction, and a 450×2 fully connected (FC) layer. Both output nodes from the FC layer were activated using the softmax function and stored as scores for the final classification task. The original images comprised three RGB channels of size 32×32×3, wherein each channel stored discrete pixels with three light intensities (red, green, and blue). Because our device demonstrated state-of-the-art sensing properties within the blue light wavelength, we only extracted pixels of the blue channel for the recognition task, which suited optical light in-memory sensing and neuromorphic visual perception.

To verify the accuracy of the D2 device-based CNN, 3822 chosen objects with the abovementioned labels from the CIFAR-10 dataset were randomly separated at a ratio of 4:1 for the model training and testing processes. The kernel value can be remotely programmed by illuminating each pixel with specific programming optical pulses. The discrete kernel values were calculated from the 16 discrete levels of the programmable states, as shown in Fig. 9b, c. Here, a pair of devices was used to cover the weight values spanning from positive to negative. As shown in Fig. 9e, both ideal convolutional kernels are offline mappings in two discrete kernels by programming the device using optical light or electrical pulses. The algorithm simulation results are shown in Fig. 9f. The software test trains on 32-bit floating-point arithmetic by default, thereby achieving the highest accuracy of 95.41%, whereas the offline mapping kernel through the D2 device obtains 91.03%, which only receives a slight decline of 4.38%.

## Conclusion

In this study, we investigated drop-cast MoS_2_-based Al/Al_2_O_3_/MoS_2_/Al_2_O_3_/P^+^-Si MOS memory devices for application in artificial visual perception and in-memory light sensing. The device showed a decent memory window of approximately 2.8 V with an operating voltage of +6/−6 V, high-temperature retention (100 °C) for 10 years, and excellent endurance (10^6^ cycles) without any deterioration. The larger threshold voltage shift with the operating voltage revealed that a greater number of electrons were trapped at the Al_2_O_3_/MoS_2_ interface and MoS_2_ layer. Interestingly, the device showed a larger shift in the memory window from 2.8 V to more than 6 V when the optical light of different wavelengths was stimulated for 2 s during the program operation. A CNN was used to measure the optical sensing and electrical programming abilities of the device. The array simulation received the optical images transmitted over the blue light wavelength and performed inference computation to process and recognize the images with 91% accuracy. The demonstrated approach is promising for the development of future artificial retina networks for artificial visual perception and in-memory light sensing applications.

## Materials and methods

### In-memory light-sensor fabrication

An MOS memory device was fabricated on a P^+^Si substrate. First, the Si substrate was wet-etched in a buffered oxide etchant for 3 min and later cleaned with deionized water and a nitrogen gun to remove the native oxide from the wafer. Furthermore, 3-nm-thick Al_2_O_3_ was used as a tunnel oxide layer by plasma-enhanced atomic layer deposition (PE-ALD) at 250 °C using Al (CH_3_), trimethylaluminum, and O_2_ plasma. Furthermore, a 70-nm-thick molybdenum disulfide (MoS_2_) film was deposited using the drop-casting method. Additionally, a 7-nm-thick Al_2_O_3_ layer was deposited by PE–ALD as a blocking oxide layer at 250 °C. Finally, a 50-nm-thick Al film was deposited as the top electrode by direct current sputtering using a metal shadow mask with a diameter of 100 µm. Spin-coated MoS_2_-based MOS devices were also fabricated for comparison purposes.

### Structural, electrical, and optical characterization of the devices

Scanning electron microscopy (SEM, Nova Nano-SEM 450) was used to examine the surface morphologies of the spin-coated and drop-cast MoS_2_ samples. The crystal structures of the spin-coated and drop-cast MoS_2_ layers were characterized using a Bruker D8 Advance X-ray diffraction (XRD) system with a Cu Kα (λ = 1.5405 Å) source at 40 kV. X-ray photoelectron spectroscopy (XPS) was performed on the drop-cast MoS_2_ sample in a high vacuum using a Kratos Amicus XPS system equipped with a monochromatic Al Kα X-ray source operating at 10 kV. Raman spectra were obtained for the drop-cast MoS_2_ sample using a Wintec Apyron Raman spectrometer equipped with a 532 nm laser source excitation. Electrical and optical measurements were performed using a Keysight B1500 A semiconductor device analyzer and tunable light source (Newport Corporation).

### Convolutional neural network design

For neuromorphic vision systems, this study was simulated based on PyTorch, which is one of the most widely used machine learning frameworks for training deep neural networks. The pattern recognition task included the binary pattern classification of images extracted from the CIFAR-10 dataset. We selected the labels “dog” and “automobile” as our classification targets for the CNN, which better exploited the device property of long-term potentiation (LTP)/long-term depression (LTD) alongside a small model.

## Supplementary information

Supplementary information
