# Supplementary material for: Artificial visual perception neural system using a solution-processable MoS2-based in-memory light sensor
Source: Light Sci Appl. 2023 May 5;12:109. doi: 10.1038/s41377-023-01166-7 (PMC10162957; doi:10.1038/s41377-023-01166-7)
Supplement: Supplementary file 1 — Supplementary information [file 41377_2023_1166_MOESM1_ESM.docx]

Supplementary Information for

**Artificial Visual Perception Nervous System Using a Solution-Processable MoS_2_-Based In-Memory Light Sensor**

Dayanand Kumar^1^, Lana Joharji^1^, Hanrui Li^1^, Ayman Rezk^2^, Ammar Nayfeh^2^, Nazek El-Atab^1^*

^1^Smart, Advanced Memory Devices and Applications (SAMA) Laboratory, Electrical and Computer Engineering Program, Computer Electrical Mathematical Science and Engineering Division, King Abdullah University of Science and Technology (KAUST), 23955, Kingdom of Saudi Arabia

^2^Department of Electrical Engineering and Computer Science, Khalifa University, Abu Dhabi 127788, United Arab Emirates

(^*^Corresponding Author: [nazek.elatab@kaust.edu.sa](mailto:nazek.elatab@kaust.edu.sa))

We extracted the property of separate devices (about 1000 devices were grown on the 2 cm x 2 cm sample using the metal mask) and conduct array level simulations. The weights of CNN are directly extracted from device conductance, which provides the possibility for future in-memory computing. We adopted method shown in Neurosim [1] to extract two device conductance and standardly normalize it as the device weights. Similar with work [2] and [3], we did array level simulation but also took device-to-device variation and cycle-to-cycle variation into consideration (Fig. S1). We first demonstrate our work with a shallow network to prove the capability of learning and potential image recognition application.

The cycle-to-cycle uniformity of the D2 was calculated with optical programming and electrical erasing for 100 continuous cycles, as shown in Fig. (a). The device depicts that the memory window is highly stable for both programming and erasing conditions. The device-to-device uniformity in both programmed and erased states of the D2 was analyzed for 20 devices which were chosen randomly, as depicted in Fig. (b). The figure confirms that the D2 shows excellent stability in both programming and erasing conditions for all 20 devices. Fig. (c) and (d) show the memory window of the D2 for 20 randomly chosen devices during optical programming (potentiation) and electrically erasing (depression), respectively. We observed the potentiation with optical programming and depression with electrical erasing shows good stability in potentiation and depression condition for all 20 devices.

Figure S1 (a). cycle to cycle uniformity of the D2 device. (b) device to device uniformity of the 20 devices which were chosen randomly. (c) Memory window of the device which is optically programmed. (d) Memory window of the device which is electrically erased

[1] Chen P Y, Peng X, Yu S. NeuroSim: A circuit-level macro model for benchmarking neuro-inspired architectures in online learning[J]. IEEE Transactions on Computer-Aided Design of Integrated Circuits and Systems, 2018, 37(12): 3067-3080.

[2] Shan X, Zhao C, Wang X, et al. Plasmonic Optoelectronic Memristor Enabling Fully Light‐Modulated Synaptic Plasticity for Neuromorphic Vision[J]. Advanced Science, 2022, 9(6): 2104632.

[3] Zhu C, Liu H, Wang W, et al. Optical synaptic devices with ultra-low power consumption for neuromorphic computing[J]. Light: Science &amp; Applications, 2022, 11(1): 1-10.
